# Sensor to detect endothelialization on an active coronary stent

**DOI:** 10.1186/1475-925X-9-67

**Published:** 2010-11-04

**Authors:** Katherine M Musick, Arthur C Coffey, Pedro P Irazoqui

**Affiliations:** 1 Weldon School of Biomedical Engineering, Purdue University, West Lafayette, IN, USA; 2 Clarian Cardiovascular Surgery, Methodist Hospital, Indianapolis, IN, USA

## Abstract

**Background:**

A serious complication with drug-eluting coronary stents is late thrombosis, caused by exposed stent struts not covered by endothelial cells in the healing process. Real-time detection of this healing process could guide physicians for more individualized anti-platelet therapy. Here we present work towards developing a sensor to detect this healing process. Sensors on several stent struts could give information about the heterogeneity of healing across the stent.

**Methods:**

A piezoelectric microcantilever was insulated with parylene and demonstrated as an endothelialization detector for incorporation within an active coronary stent. After initial characterization, endothelial cells were plated onto the cantilever surface. After they attached to the surface, they caused an increase in mass, and thus a decrease in the resonant frequencies of the cantilever. This shift was then detected electrically with an LCR meter. The self-sensing, self-actuating cantilever does not require an external, optical detection system, thus allowing for implanted applications.

**Results:**

A cell density of 1300 cells/mm^2 ^on the cantilever surface is detected.

**Conclusions:**

We have developed a self-actuating, self-sensing device for detecting the presence of endothelial cells on a surface. The device is biocompatible and functions reliably in ionic liquids, making it appropriate for implantable applications. This sensor can be placed along the struts of a coronary stent to detect when the struts have been covered with a layer of endothelial cells and are no longer available surfaces for clot formation. Anti-platelet therapy can be adjusted in real-time with respect to a patient's level of healing and hemorrhaging risks.

## Background

Coronary stents, which are routinely used to treat blocked arteries, are recognized by the body as foreign objects and can incite an immune response and cause re-occlusion of the artery. Thus, stents that elute immunosuppressive drugs have been developed that decrease this risk of re-occlusion [1,2]. These drug-eluting stents can also prevent the healing process where the stent is encapsulated in endothelial cells. The lumen is then constantly exposed to bare surfaces where clots can form at any time, even years after implantation [3]. Presently, clots are prevented pharmaceutically with clopidogrel for 12 months and aspirin indefinitely [4]. These guidelines are based on clinical averages and are not individualized based on the healing of a specific patient's stent. These drugs put the patient at risk of hemorrhaging, especially when co-administered [5].

**Figure 1:**
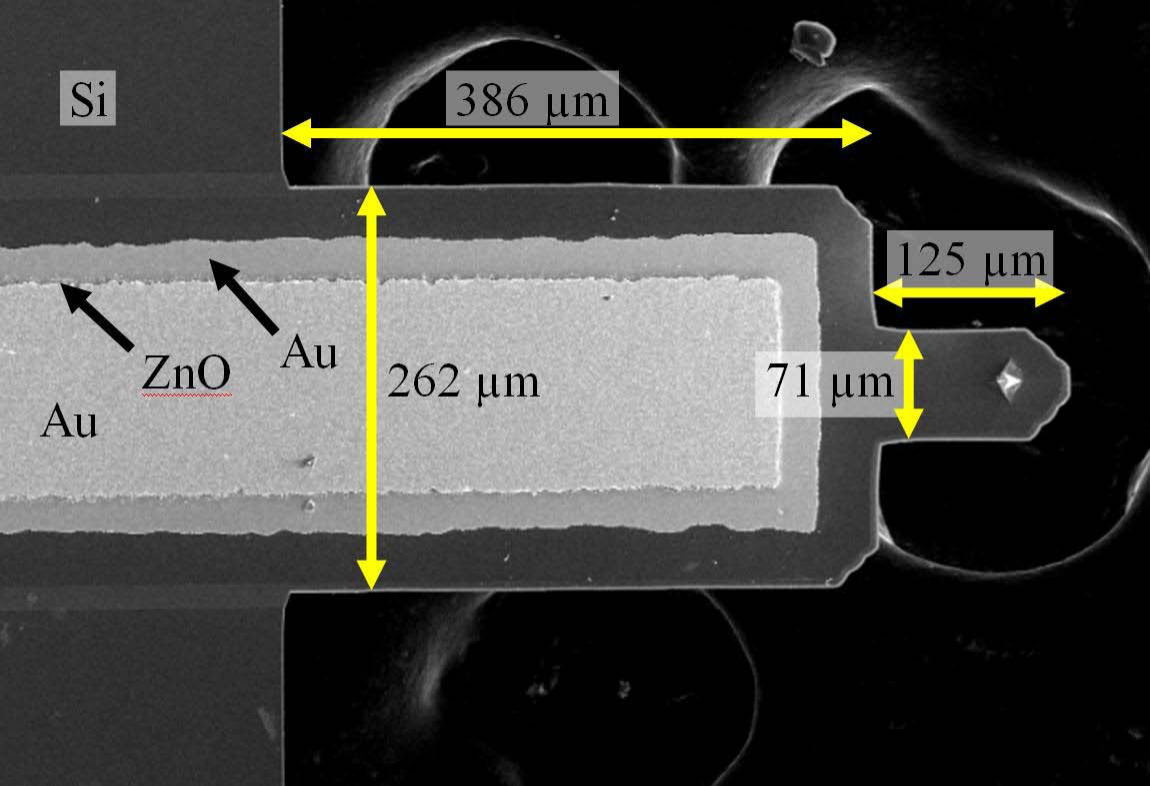
**Veeco active probe**. Cantilever consists of a Si substrate (thickness = 4 μm) supporting a ZnO stack (0.25 μm Ti/Au, 3.5 μm ZnO, and 0.25 μm Ti/Au). Other relevant dimensions are shown in microns.

Post-mortem analysis indicates that the most powerful histological predictor for late stent thrombosis is endothelial coverage, specifically, the ratio of covered to uncovered stent struts [6]. A stent that actively monitors endothelial coverage would allow physicians to better individualize a patient's anti-platelet therapy based on their clotting risk. Embedding these sensors along several struts in a stent would give detailed information regarding the level of healing in an individual patient. This article presents the development of such a sensor that consists of a commercially available piezoelectric cantilever (DMASP, Veeco Probes), which has a film of zinc oxide used to actuate the cantilever in AFM imaging applications (Figure 1). Micromachined cantilevers lend themselves well to numerous sensing applications. Attachment of molecules or whole cells onto the cantilever surface alters the effective mass and surface stress of the cantilever, and causes a shift in the cantilever's resonance frequency, as has been demonstrated previously as sensors for cell detection [7-11]. Cantilevers with integrated piezoelectric sensing elements do not require alignment of an external laser and are not affected by changes in surface reflectivity or the index of refraction of the operating fluid, allowing a more compact system. We have insulated the cantilever, allowing us to readily detect resonant frequencies in a fluid environment.

**Figure 2:**
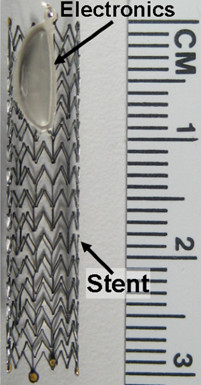
**Active stent**. Active electronics sealed in a liquid crystal polymer package and integrated with a stent.

The sensor will interface with an active stent device our lab has been developing as shown in Figure 2. By coupling a stent with a sub-mm^3 ^fully wireless implantable cardiac monitoring integrated circuit, we have created an active cardiac sensing platform which can measure pressure, flow, and oxygenation [12-14]. The stent itself is used as an antenna for wireless telemetry and powering. The sensor we have developed here will couple to this active stent to provide real-time diagnostic information regarding stent endothelial coverage without additional invasive procedures.

## Methods

**Figure 3:**
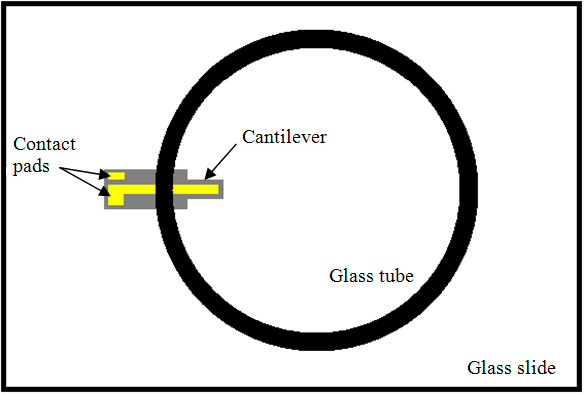
**Cell chamber**. Top view of cantilever cell chamber. Glass tube is 1 cm in diameter.

A custom chamber was devised to perform measurements in fluid (Figure 3). The fluid was confined in a glass tube (diameter = 1 cm, height = 3.5 cm) placed on top of a standard glass slide. The cantilever was placed under the rim of the tube so the cantilever itself was inside the tubing and the contact pads remained outside the tubing. The base of the tube was sealed with silicone to prevent leakage. Parylene C was deposited on the device to a thickness of 1.5 μm with a parylene CVD furnace (Specialty Coating Systems), creating water-resistant insulation on the cantilever. Parylene was selected as it is inert and should not suffer corrosion when implanted long-term. Parylene is also the primer layer on the CYPHER drug-eluting stent [15]. Thus, it should be feasible to use this cantilever in conjunction with the existing CYPHER stent so that the stent and cantilever could have similar coatings. An O_2_-plasma treatment (55 W for 30 s) effectively created a hydrophilic surface on the parylene to promote cell attachment. This step also sterilized the device.

Our structure was actuated by applying a frequency sweep from 4 to 600 kHz with an LCR meter (Agilent Technologies, E4980A) to two electrode pads contacted with micromanipulators. The voltage amplitude was set to 14 mV, though as long as the voltage is sufficiently high to minimize noise effects and well-below the published breakdown voltage of 6 V RMS, the impedance data should be independent of voltage. Actuation with the LCR meter allowed simultaneous monitoring of the impedances within the measured frequency range. It has been shown previously that frequencies with minimum impedance correspond to frequencies of maximum displacement as detected by laser vibrometry [16].

The minimum impedances are detected by searching for peaks in the plots of the impedance phase angle versus frequency. The magnitude of the impedance actually has a local maximum and minimum around the resonance, thus the phase peak does not exactly match the minimum of the admittance magnitude, but it is close and an effective measure for this work [16]. Further discussion of tracking resonant frequencies and equivalent circuit models of this type of resonator can be found in the literature [17].

The cantilever was initially characterized in cell media. Then 100 000 human coronary artery endothelial cells (Clonetics) were placed in the glass tubing for a cell density of 1300 cells/mm^2^. The sample was incubated at 37°C, 5% CO2 for 18 hrs to allow the cells to attach. The cantilevers were then re-characterized with the LCR meter to detect any changes in the resonances.

## Results and Discussion

**Figure 4:**
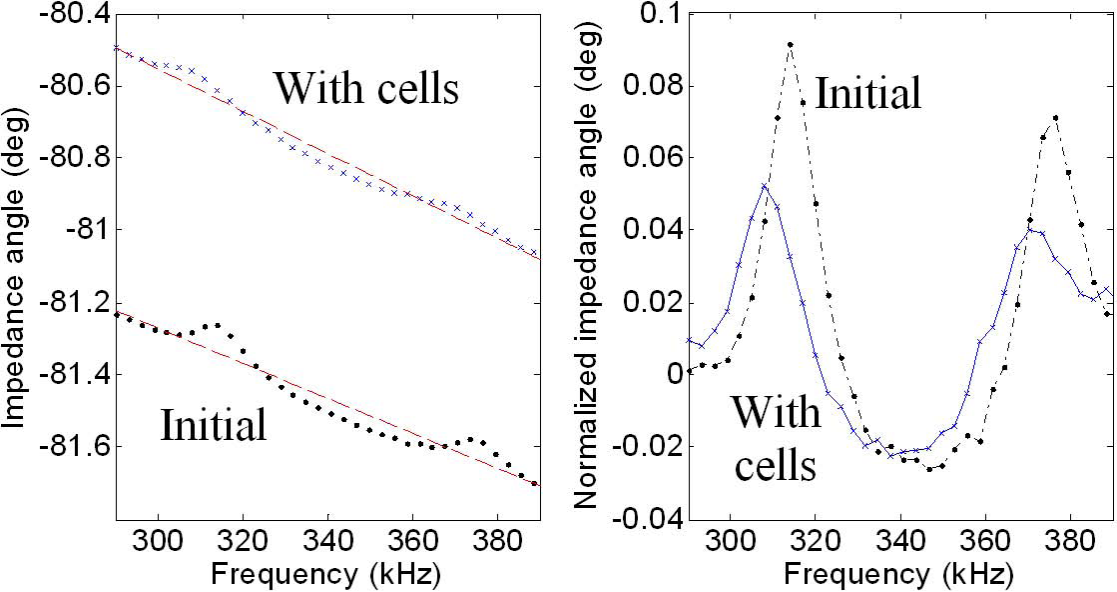
**Phase angle vs. frequency**. (left) Phase angle of impedance with linear regression line. (right) To provide an easier comparison, phase angles have been levelled with respect to the linear regression line that passes through each set of data. The addition of cells causes the peaks to shift to lower frequencies and decrease in amplitude.

Figure 4 shows the frequency response of the device before and after plating cells. In the frequency range measured (4-600 Hz), only two clear peaks can be seen in the bare device. After cells had adhered to the surface, there is a shift in both the resonant frequency and the height of these peaks. Table 1 shows the results for this trial and a second trial where the experiment was repeated to confirm the initial results. At a plating density of 1300 cells/mm^2^, there were approximately 140 cells on the portion of the cantilever free to vibrate (dimensions shown in Figure 1).

**Table 1:** Cantilever response data from two trials

	Experiment #1				Experiment #2			
	
	Freq. 1 (kHz)	Amp. 1 (deg)	Freq. 2 (kHz)	Amp. 2 (deg)	Freq. 1 (kHz)	Amp. 1 (deg)	Freq. 2 (kHz)	Amp. 2 (deg)
Initial	313.0	.086	375.5	.055	313.9	.091	376.5	.071

With cells	307.5	.031	371.0	.018	308	.052	370.5	.043

Change	5.5	.055	4.5	.037	5.9	.039	6.0	.028

The two peaks shown were the only detectable resonances despite the relatively wide frequency range measured. The electrical detection of resonances is critically compromised by the strong damping imposed by the cell media, making these measurements more challenging than those carried out in air or in a vacuum where resonance peaks show much larger amplitude [18].

In the desired in vivo application for this work, as a sensor for stent healing, it can be expected that in the course of normal healing the stent will be fully populated with a significantly thicker layer of endothelial cells. In the case of restenosis, this lining would be even thicker. This may hamper any movement of the cantilever and cause the peaks to eventually become undetectable. Frequent measurements of the resonant frequencies will differentiate this end state with the possibility of device failure. The noninvasive nature of these measurements that can be transmitted wirelessly to an external device make this an attractive and low risk option for monitoring healing.

## Conclusions

We have developed a self-actuating, self-sensing device for detecting the presence of endothelial cells on a surface. The device is biocompatible and functions reliably in ionic liquids, making it appropriate for implantable applications. This sensor can be placed along the struts of a coronary stent to detect when the struts have been covered with a layer of endothelial cells and are no longer available surfaces for clot formation. Anti-platelet therapy can be adjusted in real-time with respect to a patient's level of healing and hemorrhaging risks.

Currently, the greatest limitation of this technology is the inability to differentiate between the various cell types or any object with mass that may deposit on the surface of a stent. The possibilities for adhered masses include fibrin, clots, neointima, and endothelial cells. It has been shown that a higher ratio of stent struts covered with either neointima or endothelial cells to total stent struts is correlated with a lower incidence of late stent thrombosis [6]. In contrast, an increasing amount of fibrin on the stent surface is correlated with an increased risk [6]. Thus, the sensor must differentiate between stent coverage associated with lower incidence of thrombosis (neointima and endothelialization) and stent coverage associated with higher incidence of thrombosis (fibrin). One test that could provide this differentiation is application of a fibrinolytic drug. The sensors would be monitored as the drug was administered, if the frequency peaks indicating strut coverage persist, this would indicate that the surface is covered with substances other than fibrin or clots and thus anti-platelet therapy can be safely terminated. If the frequency peaks return to the uncoated state, the physician will be alerted that the patient is still at an increased risk of clotting and preventative measures should be continued. Future versions of this device could be designed to exploit the differences (i.e. density) of different types of biological coatings to more sophisticatedly detect stent healing.

Currently, the struts on a drug-eluting stent range from 81 to 140-μm wide [19,20]. The cantilever used in this paper is 262-μm wide. Ideally, the sensor should be thinner than the stent strut, so that it does not provide a greater surface area for potential clot formation. A similar, thinner cantilever should be developed for the final device to remedy this issue.

## Competing interests

The authors declare that they have no competing interests.

## Authors' contributions

KM constructed the prototype device, carried out the cell culture studies, electrically characterized the device, and drafted the manuscript. AC critically revised the manuscript for medical content. PI conceived of the study, participated in its design and coordination, and helped to draft the manuscript. All authors read and approved the final manuscript.
